# Cyclic Digestion and Ligation-Mediated PCR Used for Flanking Sequence Walking

**DOI:** 10.1038/s41598-020-60411-w

**Published:** 2020-02-26

**Authors:** Dong Yu, Tianshun Zhou, Xuewu Sun, Zhizhong Sun, Xiabing Sheng, Yanning Tan, Ling Liu, Ning Ouyang, Ke Xu, Kaibing Shi, Guilong Yuan, Jia Ding, Meijuan Duan, Dingyang Yuan

**Affiliations:** 1 College of Bioscience and Biotechnology, Hunan Agricultural University, 1 Nongda Rd, Changsha, 410128 People’s Republic of China; 2 State Key Laboratory of Hybrid Rice, Hunan Hybrid Rice Research Centre, 736 Yuanda Rd, Changsha, 410125 People’s Republic of China; 3 College of Agriculture, Hunan Agricultural University, 1 Nongda Rd, Changsha, 410128 People’s Republic of China; 4 Long Ping Branch, Graduate School of Hunan University, 892 Yuanda Rd, Changsha, 410125 People’s Republic of China

**Keywords:** Transgenic plants, Plant molecular biology

## Abstract

Ligation-mediated PCR (LM-PCR) is a classical method for isolating flanking sequences; however, it has a common limitation of reduced success rate owing to the circularization or multimerization of target restriction fragments including the known sequence. To address this limitation, we developed a novel LM-PCR method, termed Cyclic Digestion and Ligation-Mediated PCR (CDL-PCR). The novelty of this approach involves the design of new adapters that cannot be digested after being ligated with the restriction fragment, and cyclic digestion and ligation may be manipulated to block the circularization or multimerization of the target restriction fragments. Moreover, to improve the generality and flexibility of CDL-PCR, an adapter precursor sequence was designed, which could be digested to prepare 12 different adapters at low cost. Using this method, the flanking sequences of T-DNA insertions were obtained from transgenic rice and *Arabidopsis thaliana*. The experimental results demonstrated that CDL-PCR is an efficient and flexible method for identifying the flanking sequences in transgenic rice and *Arabidopsis thaliana*.

## Introduction

Identification of flanking sequences has often been employed to determine the location of T-DNA insertion in genomic DNA. Methods to obtain flanking sequencea include inverse PCR^1^, randomly primed PCR^2–5^, and ligation-mediated PCR^6–8^. Inverse PCR, the earliest flanking cloning technique, has a low efficiency and is limited by the rate of self-ligation and amplification range of DNA polymerases^9^. Randomly primed PCR, represented by TAIL-PCR and SiteFinding-PCR, often generates non-specific PCR products and has low reproducibility due to randomness of the primers utilized^10^. Ligation-mediated PCR includes three major steps: (i) restriction enzyme digestion of the genomic DNA and ligation of specific adapters; (ii) PCR of restriction fragments using specific primers designed from known sequences and adapter sequences; (iii) analysis of the amplified fragments using gel electrophoresis and sequencing. Owing to the use of specific primers in PCR^11,12^, ligation-mediated PCR has been one of the preferred approaches to identify flanking sequences with high sensitivity and specificity^13,14^. However, ligation-mediated PCR requires adapter ligation to generate an artificial priming site at the ends of restriction fragments. There exist common limitations due to the inherent characteristics of this method, such as reduced success rates caused by circularization or multimerization of the restriction fragment^15^, less flexibility, and high expenses, resulting in only one adapter being used with a group of restriction enzymes^14^.

Here, we report a novel method, developed based on ligation-mediated PCR and termed as cyclic digestion and ligation-mediated PCR (CDL-PCR), to circumvent the limitations mentioned above. To verify its efficiency and feasibility, CDL-PCR was used to isolate the flanking sequences of T-DNA from transgenic rice and *Arabidopsis thaliana*. The results showed CDL-PCR to be an efficient and flexible approach for isolating flanking sequences.

## Results

### Design and preparation of the adapter

**Figure 1:**
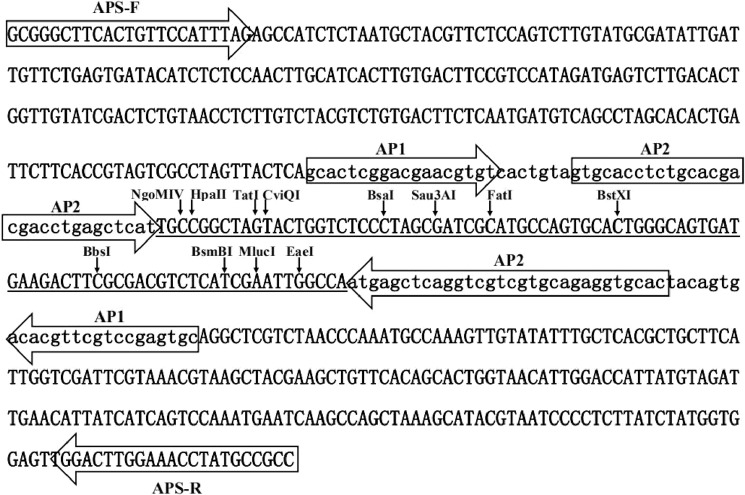
The adapter precursor sequence. Primers APS-F and APS-R were used for PCR-amplification of the adapter precursor sequence. Uppercase sequence, marked in underline, consists of 12 restriction sites, used for preparing different adapters. Two lowercase sequences are reverse-complemented, and provide nested primers AP1 and AP2 to the adapter.

To improve the compatibility and flexibility of CDL-PCR, an adapter precursor sequence (APS) was designed, which integrates 12 restriction sites between two reverse complemented sequences and includes two different sequences outside of the reverse complemented sequences for the convenience of amplification and recovery of the adapter precursor sequence (Fig. 1). The precursor sequence was synthesized and then seamlessly cloned into pMD18-T vector for the PCR template using the SSEA method^16^; the template plasmid was named pMD18T-APS. The adapter precursor sequences were enriched via PCR and digested respectively using the integrated 12 restriction sites to generate 12 kinds of adapters with different overhang ends. The adjacent bases of complementary sequences, ligated to the restriction fragment, were optimized so that every adapter could ligate to both ends of the restriction fragments digested by a group of isocaudomers, and the ligation products could no longer be cut by the same enzyme owing to the removal of the original restriction site. For example, adapter2 was generated by cutting the adapter precursor sequence with Sau3AI and the two adjacent nucleotides of the recognition site were C and G (CGATCG) (Fig. 1); therefore, no original restriction site would be produced if adapter2 ligated to the restriction fragments digested using BamHI, BglII, or BclI. The 12 adapters and corresponding isocaudomers used for digesting the genomic DNA are summarized in Table 1.

**Table 1:** Twelve adapters with different overhangs and the corresponding isocaudomers.

Adapters	Enzymes used for the preparation of adapters and their recognition sites in APS	Overhang	Corresponding isocaudomers
Adapter1	NgoMIV (G ↓ CCGG ↑ C)	5′CCGG	XmaI, AgeI, BspEI
Adapter2	Sau3AI (↓GATC ↑)	5′GATC	BamHI, BclI, BglII
Adapter3	TatI (A ↓ GTAC ↑ T)	5′GTAC	Acc65I, BsiWI, BsrGI
Adapter4	CviQI (G ↓ TA↑C)	5′TA	NdeI, AseI
Adapter5	BsaI (GGTCTCC ↓ CTAG ↑)	5′CTAG	XbaI, SpeI, NheI
Adapter6	HpaII (C ↓ CG ↑ G)	5′CG	AclI, ClaI, BstBI, NarI
Adapter7	FatI (↓CATG↑)	5′CATG	NcoI, BspHI, PciI
Adapter8	BstXI (CCAG↑TGCA ↓ CTGG)	3′TGCA	PstI, NsiI, SbfI
Adapter9	BbsI (GAAGACTT ↓ CGCG ↑)	5′CGCG	BssHII, MluI, AscI
Adapter10	BsmBI (CGTCTCA ↓ TCGA ↑)	5′TCGA	XhoI, PspXI, SalI
Adapter11	MluCI (↓ AATT ↑)	5′AATT	EcoRI, MfeI
Adapter12	EaeI (T ↓ GGCC ↑ A)	5′GGCC	NotI, EagI, PspOMI

### Protocol for cyclic digestion and ligation-mediated PCR (CDL-PCR)

**Figure 2:**
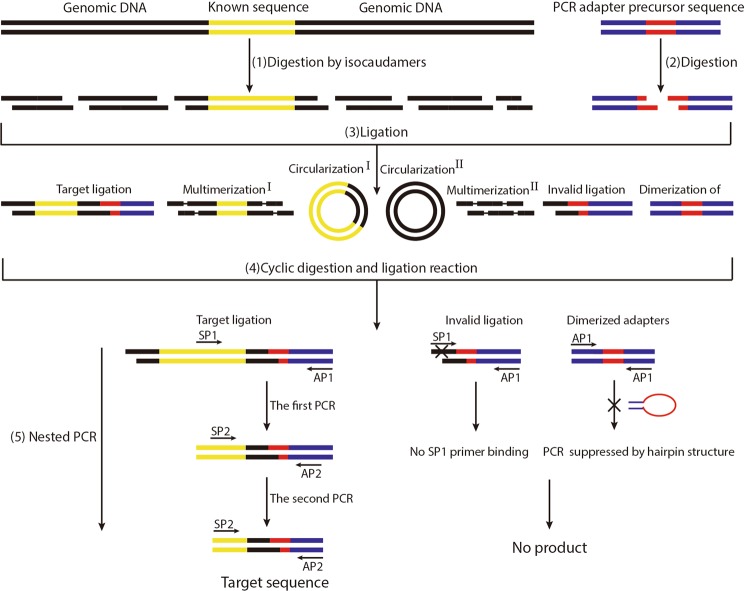
Schematic of CDL-PCR. (1) Genomic DNA was fragmented separately by a group of restriction enzymes, generating restriction fragments with same overhang ends. (2) Type I of circularization and multimerization, harboring both flanking sequence and known sequence, reduced the success rate of LM-PCR; they were cut again and ligated with adapters to form target ligation products after cyclic digestion and ligation reaction in CDL-PCR. Type II of circularization and multimerization transformed into invalid ligation, giving no product after nested PCR.

The protocol for CDL-PCR is as follows: (1) Genomic DNA was fragmented separately using isocaudomers, which belong to a group of restriction enzymes recognizing different sequences but generating restriction fragments with same overhang ends. (2) The adapter precursor sequence was amplified via PCR and an appropriate restriction enzyme was selected (based on the recognition site integrated in the adapter precursor sequence) to digest it for the preparation of adapters. (3) The restriction fragments were ligated with the adapter. Products mainly included the target ligation products formed by adapters and target restriction fragments, circularized restriction fragments, multimerized restriction fragments, invalid ligation products formed by adapters and non-target restriction fragments, and dimerized adapters. (4) Based on the compatibility of endonuclease buffer and ligase buffer, cyclic digestion and ligation manipulation were introduced to block the target restriction fragments from circularization or multimerization. Although the target ligation product could no longer be digested by isocaudomers, the circularized or multimerized target restriction fragments could be cut and ligated with the adapter to form target ligation products. (5) Nested PCR was then performed to amplify the flanking sequence adjacent to the known sequence. Figure 2 illustrates the scheme of CDL-PCR.

### Identification of T-DNA insertion sites employing CDL-PCR in transgenic rice

**Figure 3:**
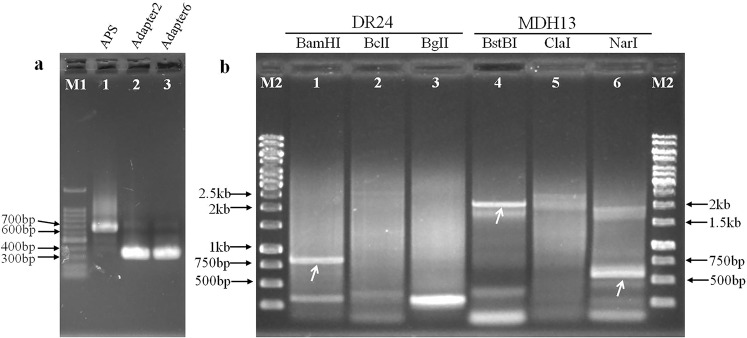
Adapter preparation and target flanking sequence amplification by CDL-PCR. M1: TAKARA 1-kb DNA ladder; M2: GeneRuler 1-kb DNA ladder; all the primers sequence used here are listed in Table 2. (**a**) Preparation of adapter2 and adapter6; lane 1 is the adapter precursor sequence (APS) amplified by PCR with primers APS-F and APS-R, lane 2 is adapter2 digested by Sau3AI, and lane 4 is adapter6 digested by HpaII; (**b**) The second round of PCR products amplified from transgenic rice DR24 and MDH13; lanes 1–3 are PCR products obtained from DR24 with primers LSP2 and AP2, lanes 4–6 are PCR products obtained from MDH13 with primers MSP2 and AP2.

**Figure 4:**
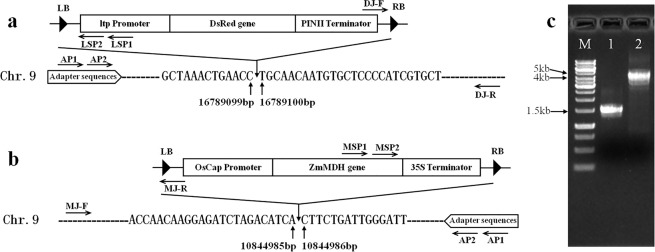
T-DNA insertion sites of transgenic rice DR24 and MDH13. LB: the left border of T-DNA; RB: the right border of T-DNA; LSP1 and LSP2 are specific nested primers designed in the upstream of ltp promoter, MSP1 and MSP2 are specific nested primers designed downstream of *ZmMDH* gene, AP1 and Ap2 are specific nested primers designed in adapter, DJ-F and DJ-R are detection primers designed according to the right border sequence and right flanking sequence of T-DNA in DR24, and MJ-F and MJ-R are detection primers designed according to the left border sequence and left flanking sequence of T-DNA in MDH13, all the primers sequence used here are listed in Table 2. (**a**) T-DNA insertion site of DR24 is between 16789099 bp and 16789100 bp on Chromosome 9; (**b**) the T-DNA insertion site of DR24 is between 10844985 bp and 10844986 bp on Chromosome 9; (**c**) The boundary PCR detection, lane 1 is PCR band about 1.5 kb with detection primers DJ-F and DJ-R and lane 2 is PCR band about 4.3 kb with detection primers MJ-F and MJ-R. Sequencing from the two bands showed that the the insertion sites of DR24 and MDH13 transgenic lines to be correct.

In a previous study, we obtained two transgenic rice lines DR24 and MDH13 with expected traits, carrying exogenous genes *DsRed* and *ZmMDH*, respectively. The two lines were planned to be subjected to an environmental release trial, which is one of the four tests for assessing GM safety in China. Therefore, it was important to get information on the T-DNA insertion sites in the rice genome; CDL-PCR was applied to retrieve the flanking sequence adjacent to the insertion sites of lines DR24 and MDH13. First, we analyzed the restriction sites in the T-DNA region of transformation vectors of the two lines. On the basis of restriction site information, we selected adapter2 and adapter6 as appropriate adapters for subsequent experiments, and designed specific nested primers LSP1/LSP2 and MSP1/MSP2 in the upstream ltp promoter sequence of *DsRed* gene and downstream sequence of *ZmMDH* gene (Table 2). Next, the APS was amplified via PCR and cut by Sau3AI and HpaII to make adapter2 and adapter6 (Fig. 3a). Genomic DNA of DR24 was separately digested by isocaudomers of BamHI, BclI, and BglII, whereas that of MDH13 was separately digested by isocaudomers of BstBI, ClaI, and NarI. The above 2 categories of restriction fragment libraries, with same overhang ends, were ligated with adapter2 or adapter6, respectively. Thereafter, cyclic digestion and ligation reactions were conducted, which translated the circularization or multimerization of target restriction fragments to the target ligation products. Nested PCR and agarose gel electrophoresis were conducted to obtain the target sequence. Finally, 3 clear bands of > 500 bp, indicated by arrows in Fig. 3b, were recovered for sequencing. The PCR band obtained from lane 1 was 803 bp and was sequenced using the primer LSP2; the bands obtained from lanes 4 and 6 were 2102 bp and 532 bp, respectively, and were sequenced using primer MSP2 and its walking primer. After sequence alignment analysis, the sequence of lane 1 contents was observed to contain partial ltp promoter sequences, vector boundary sequences, and a DNA sequence reverse complemented with Chr.9: 16788680–16789099 in the rice genome (Supplementary Fig. S1); the sequence of the lane 4 product contained part of *ZmMDH* cDNA sequence, 35 S terminator sequence, vector boundary sequences, and a DNA sequence from Chr.9: 10844986–10846153 in the rice genome (Supplementary Fig. S2); and the sequence of the lane 6 product only covered vector border sequences, with no rice genome sequence (Supplementary Fig. S3).Therefore, according to the sequencing results, the T-DNA insertion site of DR24 was between 16789099 bp and 16789100 bp on Chromosome 9 of the rice genome (Fig. 4a), and the T-DNA insertion site of MDH13 was between 10844985 bp and 10844986 bp on Chromosome 9 of the rice genome (Fig. 4b). To verify their accuracy, 2 pairs of detection primers DJ-F/R and MJ-F/R were designed on the other side of the insertion sites of DR24 and MDH13 (Fig. 4a,b). PCR detection showed the size of amplification bands to be approximately 1.5 kb and 4.3 kb (Fig. 4c), and the sequencing results proved the insertion sites of DR24 and MDH13 transgenic lines to be correct.

**Table 2:** Primers used in this study.

Primer name	Sequence of oligonucleotides (5′-3′)	Application
APS-F	GCGGGCTTCACTGTTCCATTTAG	Amplification of the adapter precursor sequence (APS)
APS-R	GGCGGCATAGGTTTCCAAGTCCA	
AP1	GCACTCGGACGAACGTGT	Nested primers in adapter
AP2	GTGCACCTCTGCACGACGACCTGAGCTCAT	
LSP1	CACCACAATGGAGGTATGTGAGGTCCGATGTACT	Specific nested primers for DR24
LSP2	GCATCCTCTTGATGAGTAAACCTCTTGAAGTACTG	
MSP1	TGATGGACGATTTTCTCTGGGAACGGAT	Specific nested primers for MDH13
MSP2	GCTTGCTGAGAAGAAATGCGTTGCCCA	
OSP1	GTAACCGACTTGCTGCCCCGAGAATTATGCAG	Specific nested primers for At-GT75
OSP2	ATGTGGGCCCCAAATGAAGTGCAGGTC	
DJ-F	CGCTCATGTGTTGAGCATATAAGAAACCCTTAG	Detection primers for DR24
DJ-R	CCGTTCTCTAGTCACTTCCACATCGATCTTC	
MJ-F	ATCTGTCTGTATCGCTCTAAGGCCCCGTTTAG	Detection primers for MDH13
MJ-R	GACTGGGAAAACCCTGGCGTTACCCAACT	
GJ4-F	GTAACCGACTTGCTGCCCCGAGAATTATGCAG	Detection primers for the insertion site on Chr. 4 of At-GT75
GJ-R	GAGTATTTCAACTGCCATTTGGGCTGAATTG	
GJ1-F	ATGTGGGCCCCAAATGAAGTGCAGGTC	Detection primers for the insertion site on Chr. 1 of At-GT75
GJ-R	GAGTATTTCAACTGCCATTTGGGCTGAATTG

### Identification of T-DNA insertion sites employing CDL-PCR in transgenic Arabidopsis thaliana

**Figure 5:**
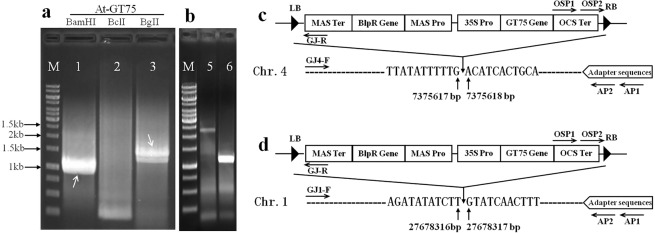
Target flanking sequence amplification and T-DNA insertion sites of transgenic *Arabidopsis* At-Gt75. M: GeneRuler 1-kb DNA ladder. LB: the left border of T-DNA; RB: the right border of T-DNA; OSP1 and OSP2 are specific nested primers (Forward primer) designed in the downstream of OCS terminator, AP1 and Ap2 are specific nested primers (Reverse primer) designed in adapter, GJ4-F and DJ-R are detection primers designed according to the right flanking sequence and the vector right boundary sequence, and GJ4-F and DJ-R are detection primers designed according to the right flanking sequence and the vector right boundary sequence; all the primers sequence used here are listed in Table 2. (**a**) The second round of PCR products amplified from transgenic *Arabidopsis* At-GT75 with primers OSP2 and AP2, 2 bands recovered from lane 1 and lane 3 are sized 1067 bp and 1411 bp by sequencing; (**b**) The right boundary PCR detection, lane 5 is a 2.3 kb PCR band with primers GJ4-F and GJ-R and lane 6 is 1.2 kb PCR band with primers GJ1-F and GJ-R. (**c**) Sequencing results from band 1and 5 showed that one of the T-DNA insertion sites in At-Gt75 was between 7375617 bp and 7375618 bp on Chromosome 4 of the *Arabidopsis* genome; (**d**) Sequencing results from band 2 and 6 showed that another T-DNA insertion site in At-Gt75 was between 27678316 bp and 27678317 bp on Chromosome 1 of the *Arabidopsis* genome.

To test whether this process was applicable in other biological systems, the T-DNA insertion sites were analyzed employing CDL-PCR in a transgenic *Arabidopsis thaliana* line At-GT75, carrying *Triticum aestivum* glycosyltransferase 75 (*GT75*) gene. The genomic DNA of At-GT75 was separately digested by isocaudomers of BamHI, BclI, and BglII, ligated with adapter2. After cyclic digestion and ligation reactions, specific primers OSP1 and OSP2 (Table 2), designed in OCS terminator, were used to perform nested PCR with primers AP1 and AP2, respectively. The two clear bands indicated by arrows in Fig. 5a, were recovered from the second round of PCR and were determined to be 1067 bp and 1411 bp, by sequencing. After alignment analysis, the 1067 bp sequence was observed to contain partial OCS terminator sequences, vector left boundary sequences, and a DNA sequence from Chr.4: 7375618–7376283 in the *Arabidopsis* genome (Supplementary Fig. S4); the 1411 bp sequence was observed to contain partial OCS terminator sequences, vector left boundary sequences, and a DNA sequence from Chr.1: 27678317–27679164 in the *Arabidopsis* genome (Supplementary Fig. S5). Therefore, one of the T-DNA insertion sites in At-Gt75 was between 7375617 bp and 7375618 bp on Chromosome 4 of the *Arabidopsis* genome (Fig. 5c) and another was between 27678316 bp and 27678317 bp on Chromosome 1 of the *Arabidopsis* genome (Fig. 5d). The right boundary PCR (Fig. 5b) and sequencing detection proved the two insertion sites of At-Gt75 to be correct. The above results showed that the transgenic *Arabidopsis* line At-GT75 carries at least two transgenic copies.

### Effectiveness of CDL-PCR

To further verify the reliability and efficiency, CDL-PCR was used to isolate the flanking sequence from the other 12 transgenic rice lines DR25-DR36, carrying the exogenous gene *DsRed*. Genomic DNA of lines DR25-DR36 were separately digested using isocaudomers of BamHI, BclI, and BglII, and subsequent operations were performed according to the CDL-PCR protocol described above. Thirteen bands of > 500 bp were recycled for sequencing from the second PCR of 12 transgenic rice lines. Among the13 bands, 7 bands (indicated by arrows in Supplementary Fig. S6) were sequenced to contain a partial ltp promoter sequences, vector boundary sequences, and a rice DNA sequence. After alignment analysis, the insertion sites of 7 transgenic rice lines were obtained and are shown in Table 3, of which DR28 and DR34 had the same insertion site and may be derived from the same transformant. The other 5 failed lines continued to be cut with another group isocaudomers of BstBI, ClaI, and NarI, and were analysis of T-DNA insertion sites by CDL-PCR. Six bands were recoveryed for sequencing, 3 bands (indicated by arrows in Supplementary Fig. S7) were sequenced to contain known sequences and a rice DNA sequence; the results of T-DNA insertion sites are shown in Table 3. Therefore, the success rate of CDL-PCR in the above 12 samples was about 58.3% (7/12) when using a group of isocaudomers and their combined efficiency was about 83.3% (10/12) when two groups of isocaudomers used together.

**Table 3:** T-DNA insertion sites of 12 transgenic rice lines DR25-DR36.

Transgenic rice lines	Isocaudomers used here	T-DNA insertion site
DR25	BamHI, BclI, and BglII	Failed
DR26	BamHI, BclI, and BglII	Chr.4: 16227727–16227728
DR27	BamHI, BclI, and BglII	Chr.8:18652264–18652264
DR28	BamHI, BclI, and BglII	Chr.6: 27858668–27858669
DR29	BamHI, BclI, and BglII	Chr.1:42534667–42534668
DR30	BamHI, BclI, and BglII	Failed
DR31	BamHI, BclI, and BglII	Chr.8: 27008930–27008931
DR32	BamHI, BclI, and BglII	Failed
DR33	BamHI, BclI, and BglII	Chr.11: 4605702–4605703
DR34	BamHI, BclI, and BglII	Chr.6: 27858668–27858669
DR35	BamHI, BclI, and BglII	Failed
DR36	BamHI, BclI, and BglII	Failed
DR25	BstBI, ClaI, and NarI	Chr.7:17617907–17617908
DR30	BstBI, ClaI, and NarI	Failed
DR32	BstBI, ClaI, and NarI	Failed
DR35	BstBI, ClaI, and NarI	Chr.12: 25858919–25858920
DR36	BstBI, ClaI, and NarI	Chr.5: 8258053–8258054

## Discussion

Information on insertion sites forms an important molecular data for the safety assessment of transgenic organisms in the world^17^ and requires a precise and efficient method for the isolation of flanking sequences. Currently, TAIL-PCR and ligation-mediated PCR are used for the identification of flanking sequences in complex genomes^17–21^, and the application of reverse PCR has become less frequent. Despite several improvements and optimizations, TAIL-PCR still has a lower success rate in practical application due to the non-specific products or short product fragments. In the beginning of the experiment, a modified TAIL-PCR, named hiTAIL-PCR^22^, was used to analyze the insertion sites of transgenic rice lines DR24 and MDH13; however, it was not successful. The classical LM-PCR, termed Loop-linker PCR^23^, was used next; however, no flanking sequence could be isolated from DR24 and MDH13 due to the success rate being reduced by circularization or multimerization of restriction fragments harboring the T-DNA. Therefore, a cyclic digestion and ligation reaction was introduced, based on the compatibility characteristics of NEB restriction enzyme and ligase buffer, to avoid circularization or multimerization of restriction fragments. The purification step after genome digestion could be omitted to simplify the entire operation. To achieve the above objective, an adapter precursor sequence was designed by nucleotide optimization, and the precursor was digested to prepare 12 different adapters with overhang ends, each of which could no longer be digested after being ligated to the restriction fragment. Furthermore, the 12 adapters could be combined with 12 groups of isocaudomers, including 35 restriction enzymes, thus greatly improving the versatility of the method and reducing its cost. Using the new and improved method, CDL-PCR, flanking sequence and insertion site information of transgenic rice and *Arabidopsis thaliana* was easily obtained.

In our experience, there are 3 key points for using CDL-PCR. First, selecting a group of isocaudomers with no corresponding restriction sites on the vector or sites farther away from the designed specific nested primers. For example, the isocaudomers of BamHI, BclI, and BgII have no corresponding restriction sites in the vector while the nearest restriction site of BstBI, ClaI, and NarI is more than 2 kb away from the specific nested primers MSP1 and MSP2. Second, some studies have suggested the cocktail of isocaudomer digestion to be able to obtain shorter restriction fragments for subsequent PCR extension^8,23^, although the shortest restriction fragment is determined by only one of the enzymes, just like the barrel theory, in which the maximum water capacity of a barrel depends on the shortest wood board. On the contrary, mixed digestion may result in insufficient enzymatic cleavage, thus influencing the final success, since the amount of a single enzyme is reduced. Therefore, separate digestion is better than mixed digestion when a group of isocaudomers are used to digest genomic DNA. Third, the success rate of CDL-PCR is limitied by the nearest distance between the restriction site and the inserted T-DNA region, if a sample failed to obtain results with one set of isocaudomers, success may be obtained with another set of isocaudomers.

In conclusion, CDL-PCR is an efficient, reliable, and cost-effective method for identifying flanking sequences in transgenic rice and *Arabidopsis thaliana*; although it is complicated to operate, it may be anticipated to be applied in more genome walking applications in the future.

## Methods

### Adapter preparation

The adapter precursor sequence was amplified from pMD18T-APS plasmid templates using Phanta Max Super-Fidelity DNA Polymerase (Vazyme Biotech) with primers APS-F and APS-R (Table 2). The PCR reactions were performed according to the manufacturer’s recommendations using the following program: 3 min at 95 °C, followed by 35 cycles of 15 s at 98 °C, 30 s at 60 °C, 50 s at 72 °C, and a final extension step of 2 min at 72 °C. According to Table 1, PCR products were digested to prepare required adapters, after purification, using the Cycle-Pure kit (Omega Bio-tek). Digested products were analyzed by 1.5% agarose gel electrophoresis, followed by recovery of the adapter, which was approximately 325-bp in size. The recycled adapters were diluted to 50 ng/μl for preservation and stand-by application.

### Construction of restriction-fragment DNA libraries and ligation with adapter

The transgenic rice lines DR24-DR36, MDH13 and At-GT75, carrying foreign genes *DsRed*, *ZmMDH*, and *GT75*, respectively, were used to identify the insertions of T-DNA. Genomic DNA was isolated from the leaves of these plants using the cetyltrimethyl ammonium bromide (CTAB) method^24^. The quality and concentration of DNA were tested using a NanoDrop 2000 spectrophotometer at 260 and 280 nm, with an OD_260/280_ range of 1.7–1.9. A group of isocaudomers (New England Biolabs), having no restriction site in the region between specific primer binding site and T-DNA border, were chosen to digest genomic DNA, the reaction condition being 1 μg genomic DNA digested overnight in 10 μl total volume using 1 μl enzymes. After complete digestion, 2 μl adapter (approximately 100 ng), 2 μl ddH_2_O, 1 μl T4 DNA ligase (New England Biolabs), and 1 μl ligase buffer were added to 10 μl restriction-fragment DNA libraries, and ligation conducted at 16 °C for 6 h in 18 μl of total volume.

### Cyclic digestion and ligation reaction

To block the circularization or multimerization of target restriction fragments, a cyclic digestion and ligation reaction was designed according to the compatibility of the buffer of NEB restriction enzyme with NEB ligase. The reaction protocol was as follows: 0.5 μl restriction enzyme, 0.5 μl restriction buffer, 0.5 μl ligase, and 0.5 μl adapter were added to 18 μl of the ligation products obtained above. The reaction program was: 10 cycles at 37 °C for 5 min, 5 min at 20 °C, and a final step of 10 min at 37 °C.

### Nested PCR amplification

To isolate flanking sequences, specific nested primers were designed based on the upstream sequence of ltp promoter, downstream sequence of *ZmMDH* cDNA and downstream sequence of OCS terminator, separately. The primers used for experiments are listed in Table 2. In the first PCR reaction, the 25 μl reaction mixture included 1 μl of the cyclic digestion and ligation product, 12.5 μl 2× Phanta Max Buffer, 0.5 μl 10 mM dNTPs, 0.5 μl AP1 primer, 1 μl LSP1 or MSP1 or OSP1primer, and 0.5 μl Phanta Max Super-Fidelity DNA Polymerase (Vazyme Biotech). The PCR program was as follows: 3 min at 95 °C, followed by 5 cycles with 15 s at 98 °C, 3 min at 72 °C, and 20 cycles of 15 s at 98 °C, 30 s at 56 °C, 3 min at 72 °C, and a final extension step of 5 min at 72 °C. In the second PCR, the 50-μl reaction mixture included 1 μl of a 20-fold dilution of the first PCR product, 25 μl 2× Phanta Max Buffer, 1 μl 10 mM dNTPs, 2.0 μl AP2 primer, 2 μl LSP2 or MSP2 or OSP2 primers, and 1 μl Phanta Max Super-Fidelity DNA Polymerase (Vazyme Biotech). The PCR cycles were as follows: 3 min at 95 °C, followed by 35 cycles of 15 s at 98 °C, 30 s at 68 °C, 3 min at 72 °C, and a final extension step of 5 min at 72 °C.

### Identification of flanking sequence

Products from the second PCR were purified with Gel Extraction kit (Omega Bio-tek) and sequenced directly by Sangon Biotech Co. Ltd. Target sequence was verified first by alignment analysis with the sequence between specific primer binding site and T-DNA border. The T-DNA flanking sequence of rice and *Arabidopsis* was analyzed with Gramene BLAST (http://www.gramene.org/). Detection primers were designed according to T-DNA flanking sequence, and the accuracy of the insertion sites was determined using PCR and sequencing.

## Supplementary information

Supplementary Dataset.
