# Supplementary material for: Cyclic Digestion and Ligation-Mediated PCR Used for Flanking Sequence Walking
Source: Sci Rep. 2020 Feb 26;10:3434. doi: 10.1038/s41598-020-60411-w (PMC7044209; doi:10.1038/s41598-020-60411-w)
Supplement: Supplementary file 1 — Supplementary Dataset. [file 41598_2020_60411_MOESM1_ESM.pdf]

Cyclic Digestion and Ligation-Mediated PCR Used for Flanking Sequence Walking

Dong Yu[1,2,+], Tianshun Zhou[2,4,+], Xuewu Sun[2,3], Zhizhong Sun[2], Xiabing Sheng[1,2], Yanning Tan[2], Ling Liu[2,4], Ning Ouyang[2,4], Ke Xu[2], Kaibing Shi[2], Guilong Yuan[2], Jia Ding[2], Meijuan Duan[3,*], and Dingyang Yuan[1,2,3,4,*]

[1]College of Bioscience and Biotechnology, Hunan Agricultural University, 1 Nongda Rd, Changsha, 410128, PRC; [2]State Key Laboratory of Hybrid Rice, Hunan Hybrid Rice Research Centre, 736 Yuanda Rd, Changsha, 410125, PRC; [3]College of Agriculture, Hunan Agricultural University, 1 Nongda Rd, Changsha, 410128, PRC; [4]Long Ping Branch, Graduate School of Hunan University, 892 Yuanda Rd, Changsha, 410125, PRC

*Correspondence and requests for materials should be addressed to D.Y. (yuandingyang@hhrrc.ac.cn) or M.D. (duanmeijuan@163.com)

[+]Dong Yu and Tianshun Zhou contributed equally to this work.

Supplementary information

1bp

ACCACCACATTTTATTTATCCTCATCGGCTTATTTTTAGGCCACGGTTATTCTCACGAAGAGACGGTTTAATCG

TCGACCTGCAGGCATGCAAGCTTGGCACTGGCCGTCGTTTTACAACGTCGTGACTGGGAAAACCCTGGCGTTAC

CCAACTTAATCGCCTTGCAGCACATCCCCCTTTCGCCAGCTGGCGTAATAGCGAAGAGGCCCGCACCGATCGCC

CTTCCCAACAGTTGCGCAGCCTGAATGGCGAATGCTAGAGCAGCTTGAGCTTGGATCAGATTGTCGTTTCCCGC

Chr.9:16789099

CTTCAGTTTAAACTATCAGTGTTTGAAAAGTGGTGGTAAAAGTGGTggttcagtttagcttttgcgttggtggg

ctaaagcatgtttcttgccatcaaagctagacatgaatcattcaggatgttagttggatgcttccaaccagaaa

ccatggctagcttctcccttctcccatggatgcatgcaaacgtggagtaggaataggatagtagtagcagcagc

agtgtccacagtgtagggaagcccctttcctaaaggcctttcatgacaccaccaagtgggcccctccaagcag

gccatgtgaatagctcatcttttggtggtcacttgccagtttagtagcactgttacctgataggtacttaactt

agtcattagacattaggtgcaacccttaagctggtcatcaagcctctcctgctctaagccactgacataataat

Chr.9:16788680

cccccagttgaagaaactatcttatgaagatgagttgaacttggaaccataaaattcatgggc

803bp

Supplementary Figure S1. Alignment analysis of an 803-bp target sequence obtained from DR24. The red sequence represents partial ltp promoter sequence, the blue sequence represents left-border vector sequences and the black and lowercase sequence represents flanking sequence in rice genome from Chr.9: 16789099–16788680.

1bp

ATGCATTCTGTGATCTTCCGGAAGATACCATGCTACCAGGAGAAGTGTAGGGTACCGAGCTCGAATTCGACCGGATCTGTCGATCG
ACAAGCTCGAGTTTCTCCATAATAATGTGTGAGTAGTTCCCAGATAAGGGAATTAGGGTTCCTATAGGGTTTCGCTCATGTGTTGA
GCATATAAGAAACCCTTAGTATGTATTTGTATTTGTAAAATACTTCTATCAATAAAATTTCTAATTCCTAAAACCAAAATCCAGTA
CTAAAATCCAGATCCCCCGAATTAATTCGGCGTTAATTCAGTACATTAAAAACGTCCGCAATGTGTTATTAAGTTGTCTAAGCGTC
AATTTGTTTACACCACAATATATCCTGCCACCAGCCAGCCAACAGCTCCCCGACCGGCAGCTCGGCACAAAATCACCACTCGATAC
AGGCAGCCCATCAGTCCGGGACGGCGTCAGCGGGAGAGCCGTTGTAAGGCGGCAGACTTTGCTCATGTTACCGATGCTATTCGGAA
GAACGGCAACTAAGCTGCCGGGTTTGAAACACGGATGATCTCGCGGCCCACATACAAATGGACGAACGGATAAACCTTTTCACGCC
CTTTTAAATATCCGATTATTCTAATAAACGCTCTTTTCTCTTAGGTTTACCCGCCAATATATCCTGTCAAACACTGATAGTTTAAA
CTGAAGGCGGGAAACGACAATCTGATCCAAGCTCAAGCTGCTCTAGCATTCGCCATTCAGGCTGCGCAACTGTTGGGAAGGGCGAT
CGGTGCGGGCCTCTTCGCTATTACGCCAGCTGGCGAAAGGGGGATGTGCTGCAAGGCGATTAAGTTGGGTAACGCCAGGGTTTTCC

Chr.9:10844986

CAGTCACGACGTTGTAAAACGACGGCCAGTGCCAAGcttctgattgggattaaggtaatgagccctatctgatgtcagtggggatt
gtttacagtaccgctgatgcactagcaaacatagacgtatgggcctggacccatatgttagccaccgctactgcatcagcagtatt
gcagaggatttgcatccgcctagatggttcctaggagcataggagtacagatgggggcgcacaaagccgggtcagtttacccaact
accttcctcctcttaactataacttatatttaatttatgtctctcgaaaatagatatgaacatactttttttaaaaaataatactat
atattgtgaatttgtgttccttacctttacatttgagttatgacgaacaactttatcgattatataaaagaaaggatgacttctta
tccaaacaaatcctatagtaatgtcttttaactttcagtgactaacatataaaccatcaaacgagtccatattgaaggataatact
acgaagaatagtcatcccacatttttacactgccactatcagttaaaactgaaaaccagctcaccccaagctcaccaagaatcttc
gagaaacttataaactccgccgaaaaatctcggacaaacccgcggctcacacgcctccacgcacccaaaccccaccctagaatatc
ctctccttggccaccgcgccgccacatcagcctccccaatctccccgccccacgcgcgagcgccaatcgcgagccgcctttagatt
tcccaagataaggactcgatccccccctcacttcccgcgctatttaaactcccgcgccatctccaactcccaactcacactcgctcg
ctcatcgccatctctctcagctctcacagctcactgcatcaatggccgcggccaccatggcgctctcctccccggtgatggcccgc
gcggcgccgtcgacctcctccgcgctcttcggcgaggcgcggatcaccatgcgcaagaccgccgcgaagcccaagccggcggcgtc
gtcggggagcccgtggtacggcgccgaccgcgtcctctacctcggcccgctctccggcgagccgccgagctacctcaccggcgagt
tcccgggcgactacgggtgggacaccgcggggctctccgccgacccggagacgttcgccaagaaccgggagctggaggtgatccac

Chr.9:10846153

tcccggtgggcgatgttgggcacgctcggccgcgtctt

2102bp

Supplementary Figure S2. Alignment analysis of a 2102-bp target sequence obtained from MDH13. The red sequence represents a part of *ZmMDH* cDNA sequence, the yellow sequence represents 35S terminator sequence, the blue sequence represents right-border vector sequence, and the black and lowercase sequence represents flanking sequence in rice genome from Chr.9: 10844986–10846153.

1bp
↓
GAGAGGGATGCATTCTGTGATCTTCCGGAAGATACCATGCTACCAGGAGAAGTGTAGGGTACCGAGCTCGAATT
CGACCGGATCTGTCGATCGACAAGCTCGAGTTTCTCCATAATAATGTGTGAGTAGTTCCCAGATAAGGGAATTA
GGGTTCCTATAGGGTTTCGCTCATGTGTTGAGCATATAAGAAACCCTTAGTATGTATTTGTATTTGTAAAATAC
TTCTATCAATAAAATTTCTAATTCCTAAAACCAAAATCCAGTACTAAAATCCAGATCCCCCGAATTAATTCGGC
GTTAATTCAGTACATTAAAAACGTCCGCAATGTGTTATTAAGTTGTCTAAGCGTCAATTTGTTTACACCACAAT
ATATCCTGCCACCAGCCAGCCAACAGCTCCCCGACCGGCAGCTCGGCACAAAATCACCACTCGATACAGGCAGC
CCATCAGTCCGGGACGGCGTCAGCGGGAGAGCCGTTGTAAGGCGGCAGACTTTGCTCATGTTACCGATGCTATT
CGGAAGAACGGCAA
↑
532bp

Supplementary Figure S3. A 532-bp sequence, obtained from MDH13 alignment analysis, only covered vector border sequence but no rice genome sequence. The red sequence represents *ZmMDH* cDNA sequence partially, the yellow sequence represents 35S terminator sequence, and the blue sequence represents vector right border sequence.

1bp
↓
CGACAAATCGTTGGGCGGGTCCAGGGCGAATTTTGCGACAACATGTCGAGGCTCAGCAGGACCTGCAGGCATGCAAGCTTGGCACT
GGCCGTCGTTTTACAACGTCGTGACTGGGAAAACCCTGGCGTTACCCAACTTAATCGCCTTGCAGCACATCCCCCTTTCGCCAGCT
GGCGTAATAGCGAAGAGGCCCGCACCGATCGCCCTTCCCAACAGTTGCGCAGCCTGAATGGCGAATGCTAGAGCAGCTTGAGCTTG
GATCAGATTGTCGTTTCCCGCCTTCAGTTTAAACTATCAGTGTTTGACAGGATATATTGGCGGGTAAACCTAAGAGAAAAGAGCGT
Chr.4:7375618
↓
TTATTAGAATAACGGATATTTAAAAGGGCGTGAAAAGGTTTATCCGTTCGTCCATTTGacatcactgcacacatggtgagatattg
tgtgcgaaagactcttagacgaatgaaagaattcaacttttcccatgaacttattcgagctactcacatgtgtgtgacatatagtt
tttgatagttacaaattgttaggtcgttagacttggtaattctacttggttagttatgttttcctttcaaaataattcagagttcc
tattcttcatctctacaaaaattcagctgtcgagagtgaagaatcaactttaattcaacgactgtttcggtaagatattcgttttt
gaatcaacatattgttagaatcaacctagagaagaatcaacatacagtaatttttattttcttaaaaaatatttttagttgaggt
agtggtatggggtaaatattttgagaattaaacaaataaaaagaagtattaaatagtttttaaaacacaaaaataggtttaagaa
atttggaaaaaaaataaaatatatcacaaaattgttctctttataagtaactcgtaacgatgccttctctttcattctctgcatta
actaactccactctttaacaggaagagaccagaggaattccaaatttctattagattcacattaactttcaaatatttccttttg
Chr.4:7376283
↓
ggttcttcgagtgtgttgaaatgtcgagaacgaag
↑
1067bp

Supplementary Figure S4. Alignment analysis of the target sequence size of 1067 bp obtained from At-GT75. The red sequence represents a partial OCS terminator sequence, the blue sequence represents left-border vector sequences, and the black and lowercase sequence represents the flanking sequence in the *Arabidopsis* genome from Chr.4: 7375618–7376283.

1bp

TGACGACAAATCGTTGGGCGGGTCCAGGGCGAATTTTGCGACAACATGTCGAGGCTCAGCAGGACCTGCAGGCATGCAAGCTTGGC
ACTGGCCGTCGTTTTACAACGTCGTGACTGGGAAAACCCTGGCGTTACCCAACTTAATCGCCTTGCAGCACATCCCCCTTTCGCCA
GCTGGCGTAATAGCGAAGAGGCCCGCACCGATCGCCCTTCCCAACAGTTGCGCAGCCTGAATGGCGAATGCTAGAGCAGCTTGAGC
TTGGATCAGATTGTCGTTTCCCGCCTTCAGTTTAAACTATCAGTGTTTGACAGGATATATTGGCGGGTAAACCTAAGAGAAAAGAG
CGTTTATTAGAATAACGGATATTTAAAAGGGCGTGAAAAGGTTTATCCGTTCGTCCATTTGTATGTGCATGCCAACCACAGGGTTC
CCCTCGGGATCAAAGTACTTTGATCCAACCCCTCCGCTGCTATAGTGCAGTCGGCTTCTGACGTTCAGTGCAGCCGTCTTCTGAAA

Chr.1:27678317

ACGACATGTCGCACAAGTCCTAAGTTACGCGACAGGCTGCCGCCCTGgtatcaactttcatcttctgatattcaccaaaacttcta
tccaaaaccaagttcaaaaatatgaattgcctcatgttcttcaaaatgctattagcattctctttcattagtctattttatgttgg
taatgcacaacaatcatatggcaattcaacggtttcggcacttttcgcttttggagactcaatacttgacacaggcaacaataatc
ttcttttaagtgtttctaaagtcaatttttatccatacggtagagactttataggtggaagagctactgggagatttggaaatggg
agagtttttctgatataattggtatgtatatatatattgatctctacttgttgatatacttgtaacggatgaattcaatgtttgt
aatactatgacacaaatattatctaatagcaagtctttgttttcagctgagggtttgggattgaagaatctcttaccagcatatcg
tgatccatacctctggaacaatgatctaacgactggtgtttgtttcgcatctggtggatctggacttgatccaatcactgcaagaa
caacagtaaatttttcttagtttgtccaaaagattataacaacttttattttatctatagcaaattttaatagattatattttggt
gtgattagggaagtatatgggtgtcagaccaggtcacagacttccaaaactacatcaccagactaaatggtgtagtaggaaatcaa
gaacaagcaaatgcagttatatcaaatgctgtttacctaatctccgcaggaaataatgatattgctatcacttattttactacggg

Chr.1:27679164

ggctagaagattacaatacactcttccagcctaca

1411bp

Supplementary Figure S5. Alignment analysis of the target sequence size of 1411 bp obtained from At-GT75. The red sequence represents a partial OCS terminator sequence, the blue sequence represents left-border vector sequences and the black and lowercase sequence represents the flanking sequence in the *Arabidopsis* genome from Chr.1: 27678317–27679164.

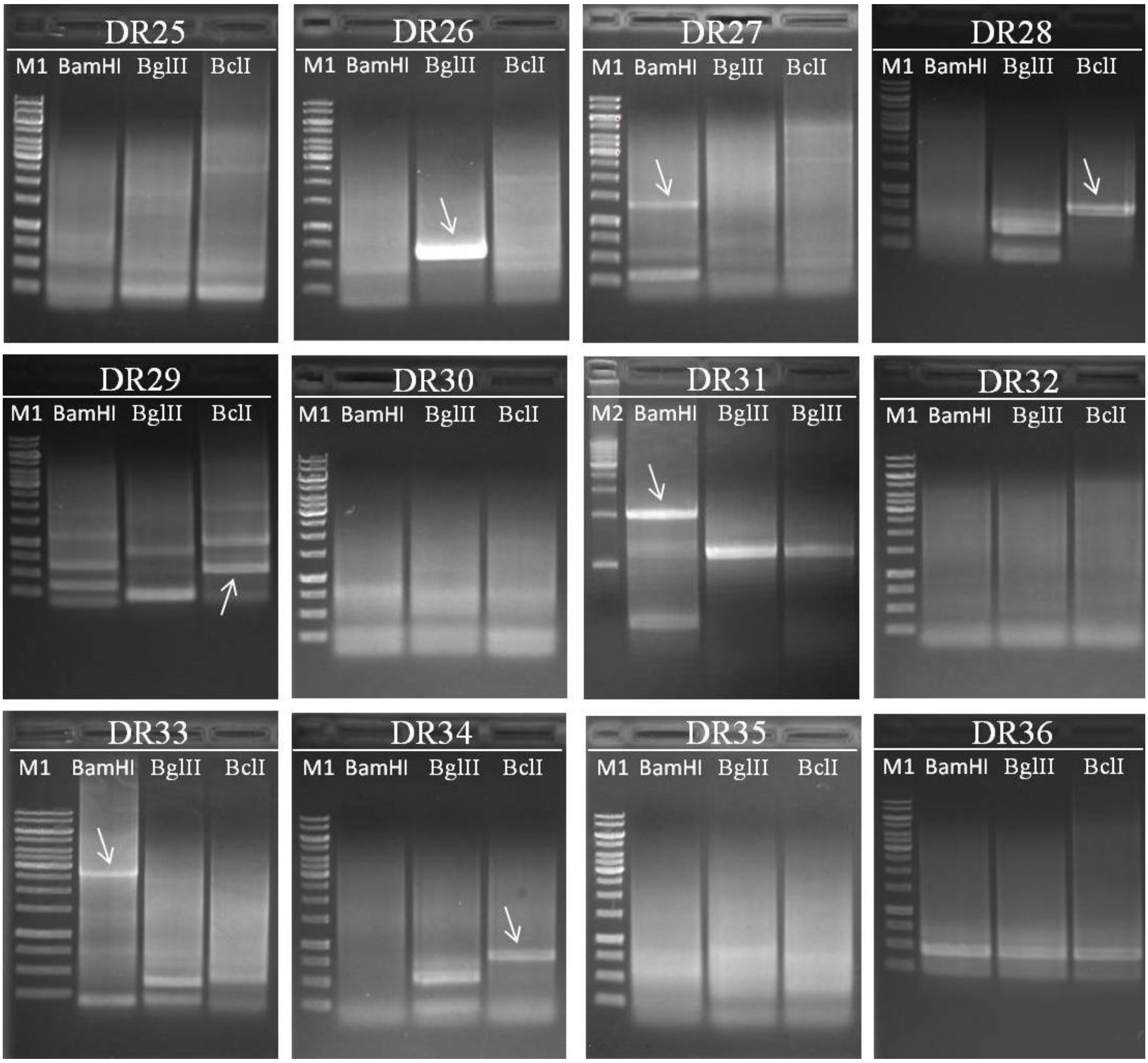

Supplementary Figure S6. The second round of PCR products amplified from 12 transgnic rice lines. DR25-DR36 restriction-fragment DNA libraries were generated by BamHI, BclI, and BglII. 13 bands of > 500 bp were recycled for sequening, Seven corrected insertion sites were identified from the sequencing results of the bands indicated by arrows, of which DR28 and DR34 had the same insertion site. Flanking sequences were not obtained for the 5 lines of DR25, DR30, DR32, DR35, and DR36.

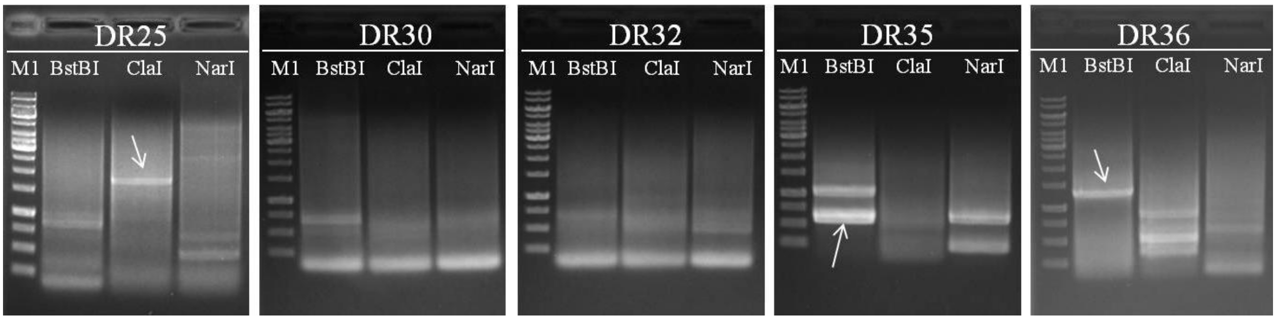

Supplementary Figure S7. The second round of PCR products amplified from DR25, DR30, DR32, DR35 and DR36. Restriction-fragment DNA libraries were generated using BstBI, ClaI, and NarI. Three corrected insertion sites were obtained from the sequencing results of the bands indicated by arrows. Flanking sequences were not obtained for DR30 and DR32.

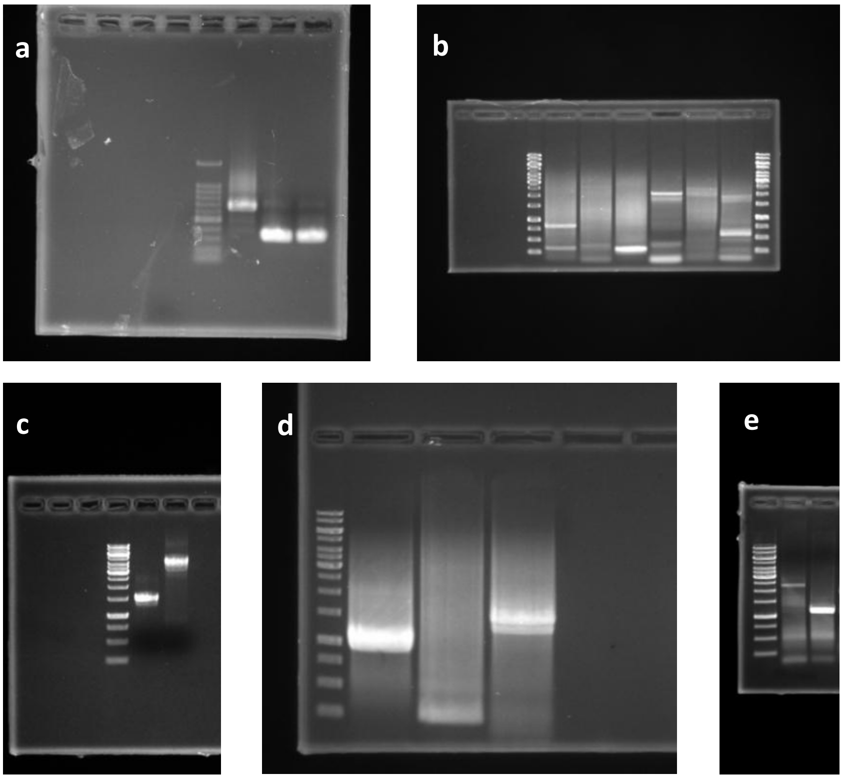

Supplementary Figure S8. (a) Full-length gels of Figure 3a; (b). Full-length gels of Figure 3b; (c) Full-length gels of Figure 4c; (d) Full-length gels of Figure 5a; (e) Full-length gels of Figure 5b.
